# Assessment of the Common Risk Factors Associated with Type 2 Diabetes Mellitus in Jeddah

**DOI:** 10.1155/2014/616145

**Published:** 2014-09-01

**Authors:** Manal A. Murad, Samia S. Abdulmageed, Rahila Iftikhar, Bayan Khaled Sagga

**Affiliations:** ^1^Department of Family and Community Medicine, King Abdulaziz University, P.O. Box 42806, Jeddah 21551, Saudi Arabia; ^2^Department of Public Health Nursing, King Abdulaziz University, Saudi Arabia; ^3^ Health Promotion Management Master's Program, College of Arts and Sciences, American University, Washington, DC, USA

## Abstract

Risk factor management is important in avoiding life-threatening complications and preventing new-onset diabetes. We performed a case-control study in 2013 at ten primary health care centers in Jeddah, Saudi Arabia to determine the common risk factors of diabetes mellitus type 2 (DM2) and the demographic background of adult Saudi patients with DM2. Known diabetic patients were recruited as cases, while nondiabetic attendants were selected as controls. A pretested designed questionnaire was used to collect data from 159 cases and 128 controls. Cases were more likely than controls to be men (*P* < 0.0001), less educated (*P* < 0.0001), natives of eastern Saudi Arabia (*P* < 0.0001), retired (*P* < 0.0001), lower-salaried (*P* < 0.0001), or married or divorced (*P* < 0.0001). By univariate analysis cases were likely to be current smokers (*P* < 0.0001), hypertensive (*P* < 0.0001), or overweight/obese (*P* < 0.0001). Cases were also more likely to have a history of DM in a first-degree relative (*P* = 0.020). By multivariate analysis, cases were more likely to be older than 40 years (*P* < 0.0001), less educated (*P* = 0.05), married or divorced (*P* = 0.04), jobless/housewives (*P* < 0.0001), or current smokers (*P* = 0.002). They were also more likely to have salaries <7000 Saudi riyals (*P* = 0.01). Overall, prediabetic and high risk groups should be identified and counseled early before the occurrence of diabetes.

## 1. Background

Diabetes mellitus type 2 (DM2) is a metabolic disorder of multiple etiologies due to disturbances of carbohydrate, fat, and protein metabolism. It is characterized by chronic hyperglycemia, and it is associated with cardiovascular and renal complications [1]. These complications result in diminished quality of life and reduced life expectancy. In addition, the disease places a considerable economic burden on worldwide healthcare resources [2]. The estimated number of deaths due to diabetes is similar to the combined number of deaths from several infectious diseases such as human immunodeficiency virus (HIV)/AIDS, malaria, and tuberculosis [3].

In 2011, it was estimated that 366 million people were diabetic. The expected prevalence of diabetes in 2030 is 366 million, and approximately 530 million people may be diabetic by 2030 [4]. Up to three Arabic-speaking countries, including Saudi Arabia, Kuwait, and Qatar, are among those with the highest prevalence of DM2 [5]. In 2004, a national survey revealed that the prevalence of DM among adults in Saudi Arabia was 23.7%, which is substantially very high [5]. A recent study showed that the prevalence of DM2 in Saudi Arabia was 17.7% and 16.4% in men and women, respectively [6]. Hence, diabetes is a serious public health problem in Saudi Arabia, as approximately one out of five Saudis is diabetic [7].

Although DM2 is associated with complications, it is a preventable disease. Morbidity and mortality can be reduced by secondary prevention through regular screening, early detection of DM and its complications, and appropriate treatment of chronic complications. To control DM, it is necessary to determine associated risk factors. Uncontrollable factors include socioeconomic status, age, sex, genetic susceptibility, and other environmental factors. Controllable risk factors include obesity [8], hypertension [9], dyslipidemia [10], and smoking [11]. It is very important to manage these risk factors to prevent or delay the onset of DM2 as well as avoid the occurrence of life-threatening complications. Despite this, a high proportion of patients with risk factors for diabetes-related complications are not adequately controlled [12]. Therefore, improvements in disease management and monitoring are required to ensure that guideline targets are met [13].

In order to set a program for the screening of DM2 in the prediabetic stage or earlier, it is necessary to define recent risk factors associated with diabetes. To the best of our knowledge, no study has assessed the risk factors for diabetes among low- and middle-income Saudi patients with DM2. Hence, the primary objective of this study was to determine the common risk factors associated with DM2 and the demographic background of adult Saudi patients with DM2 as well as the role of these factors in the development of complications. We also aimed to determine the impact of dietary and nondietary control among diabetic patients. We believe that the results of this study will help healthcare administrators to improve health education and design a community-directed strategy for DM2.

## 2. Methodology 

A case-control study was performed at diabetes care centers in Jeddah in 2013. Adult Saudis of both genders who were known diabetic patients were recruited as cases if they had fasting blood glucose levels ≥126 mg/dL (7 mmol/L) or were on hypoglycemic drugs or insulin [3]. Nondiabetic Saudi attendants of the primary health care centers (PHCs) were selected as controls.

### 2.1. Recruitment of Participants

Jeddah is divided into 44 PHCs, which cover four geographical sectors, namely, north, south, east, and west. The ten centers have specialized diabetic care clinics, which are distributed in the four sectors.

To recruit participants, sampling was done in two stages. First, a representative sample of the ten PHCs was selected because they had specialized diabetic clinics. The ten centers are not distributed equally across the four geographical sectors; they are rather distributed according to the population density of the sector. In the second stage, participants were selected based on the days that diabetic patients were scheduled for visits and days that clinics were open for patient follow-up.

We developed a computer-generated simple random sample from each center.

Eligible patients meeting study criteria who consented to participate were recruited. Patients who withdrew from the interview and/or pregnant women were excluded from the study. Ethical clearance was obtained from the Research Ethics Committee of the Directorate of Health Affairs of Jeddah.

### 2.2. Sample Size

The estimated prevalence of DM2 in Saudi Arabia is approximately 24% [5]. To calculate the sample size, we assumed an alpha error of 0.05 and a 95% confidence level, an allocation ratio of controls to cases of 1, and a hypothetical proportion of cases with exposure of 24. Using these parameters, 159 cases and 128 controls were required to detect the least extreme odds ratio of 0.32 [14]. A margin of 45% was added to guard against any refusal or missing data.

### 2.3. Data Collection Techniques and Tools

A face-to-face interview was administered to all the participants using a pretested designed questionnaire that was adapted from the World Health Organization STEPwise approach to chronic disease risk factor surveillance (STEPS) [15]. A data collecting sheet was used for extracting data from the medical records of the participants; the most recent laboratory results were recorded for both cases and controls.

## 3. Results

We recruited 159 cases and 128 controls. The duration of diabetes was as follows: <5 years in 41.4% of the patients, 6–10 years in 28.7% of the patients, 11–15 years in 15.3% of the patients, and >15 years in 14.6% of the patients.  Table 1 shows the distribution of factors associated with DM, while Table 2 shows the results of univariate analysis of the factors associated with DM. When compared with controls, cases were more likely to be men, less educated (*P* < 0.0001), natives of eastern Saudi Arabia (*P* < 0.003), unemployed/housewives, retired, or less salaried (*P* < 0.0001). Diabetic patients were also more likely to be either married or divorced and had a history of diabetes in a first-degree relative (mother, father, brother, or sister). Amongst the women, 17.7% of the cases had a history of gestational diabetes compared with 11.9% of the controls.

### 3.1. Hypertension and Diabetes

Diabetic patients were more likely to have hypertension compared with nondiabetic patients (*P* < 0.0001). Hypertension was diagnosed prior to the diagnosis of diabetes in 33.3% (*n* = 24) of the patients, whereas diabetes was diagnosed before hypertension in 43.1% (*n* = 31) of the patients; hypertension and diabetes were diagnosed concurrently in 23.6% (*n* = 17) of the cases.

### 3.2. Body Weight and Diabetes

Cases were more likely to have a body mass index (BMI) ≥ 25 kg/m^2^ (*P* < 0.001). Nevertheless, there was no significant association between activity level and diabetes. Patients who were physically active, those who did not perform household chores, and those who had a servant were more likely to have DM2; however, these results were not statistically significant. Similarly, there was also no significant association between diabetes and the patients' self-perception of being physically active (Table 2).

### 3.3. Thyroid Disease and Diabetes

Fifteen of the patients (10.1%) had hypothyroidism as against 80.5% (*n* = 120) who did not have hypothyroidism; 9.4% of the patients (*n* = 14) did not know whether they had a thyroid disorder.

### 3.4. Dietary Patterns

*(a) Consumption of Sugar and Sweets*. Approximately 8.2% of the cases used more than four teaspoons of sugar as compared with 11.2% of the controls; 14.7% of the cases consumed sugar daily as compared with 37.3% of the controls. Regarding the frequency of consumption, 19.2% of the cases as against 19.0% of the controls consumed sweets three to six times weekly; 38.5% of the cases versus 34.1% of the controls consumed sweets <3 times weekly.

Conversely, 27.5% of the cases as against 9.5% of the controls did not consume sweets. Among the cases, approximately 6.4% consumed soft drinks daily, 5.8% consumed soft drinks 3–6 times weekly, and 34.0% drank soft drinks <3 times weekly; 53.8% did not consume soft drinks. On the other hand, 17.5%, 17.5%, and 30.2% of the controls drank soft drinks daily, 3–6 times weekly, and <3 times weekly, respectively; 34.9% of the controls did not consume soft drinks.

*(b) Consumption of Fatty Foods*. The frequency of consumption of fatty foods differed among the cases: 15.5% ingested fatty foods daily, 24.3% consumed fats 3–6 times weekly, and 35.8% had fatty foods <3 times weekly. Only 24.3% of the cases did not consume fried or fatty foods. In comparison, 28%, 44%, and 47% of the controls consumed fatty foods every day, 3–6 times per week, and <3 times per week, respectively; 8% of the controls did not consume fried or fatty foods.

*(c) Starch Consumption*. Among the cases, 42.2% consumed starch every day, 25.3% consumed starch 3–6 times per week, and 26.6% consumed starch <3 times per week; 5.8% of the cases had starch-free diets. In comparison, 66%, 31%, and 24% of the controls consumed starch daily, 3–6 times per week, and <3 times per week, respectively; 4% of the controls had starch-free meals.

*(d) Smoking Status*. Thirty-six of the 159 cases (22.6%) smoked as compared with six of the controls (4.7%; *P* < 0.003). Among the smokers, all of the six patients (100.0%) in the control group smoked cigarettes, while 57.7% of the cases smoked cigarettes. On the contrary, 42.3% of the cases smoked shisha, while none of the six patients in the control group smoked this form of tobacco. Regarding smoking duration, 23.8%, 9.5%, and 66.0% of the cases had smoked for <5 years, >5 years, and >10 years, respectively. On the other hand, 20%, 40%, and 40% of the controls had smoked for <5 years, >5 years, and >10 years, respectively. None of the cases had tried to quit smoking as compared with 16.7% of controls.

### 3.5. Healthy Dietary Patterns

*(a) Sugar Consumption*. We found that 44.7% of the cases consumed sugar regularly in tea and coffee as compared with 75.6% of the controls. Among participants who consumed sugar regularly, 67.0% of the cases as compared with 42.1% of the controls consumed <2 teaspoons of sugar daily. On the contrary, 24.7% of the cases versus 46.7% of the controls consumed 2–4 teaspoons of sugar.

*(b) Consumption of Vegetables*. Among the cases, 39.6% consumed vegetables daily, 29.2% consumed vegetables 3–6 times weekly, and 21.4% had vegetables <3 times weekly; 9.7% of the cases did not consume vegetables. On the contrary, 34.6%, 24.4%, and 27.6% of the controls consumed vegetables daily, 3–6 times weekly, and <3 times weekly, respectively; 13.4% of the controls did not consume vegetables.

*(c) Fruit Consumption*. Approximately 47.1% of the cases consumed fruits every day; 24.8% of the cases consumed fruits 3–6 times per week, while 24.8% had fruits <3 times per week. About 3.2% of the cases did not consume fruits. In comparison, 30.6%, 26.6%, and 35.5% of the controls had fruits every day, 3–6 times per week, and <3 times per week, respectively; 7.3% of the controls did not consume fruits.

*(d) Grains*. Among the cases, 38.7% consumed grains daily; 21.3% consumed grains 3–6 times weekly, while 20.6% had grains <3 times weekly. About 19.4% of the cases did not consume grains. In comparison, 16.1%, 18.5%, and 37.1% of the controls had grains daily, 3–6 times weekly, and <3 times weekly, respectively; 28.2% of the controls did not consume grains.

#### 3.5.1. Blood Sugar, Blood Pressure, and Cholesterol Readings

Table 3 shows the mean weight, height, blood glucose, blood pressure, mean serum cholesterol, low-density lipoprotein (LDL), and high-density lipoprotein (HDL) of the cases and controls.

#### 3.5.2. Personal Care of Diabetes

*(a) Regular Medicines*. A large proportion of cases (93.6%) took their medicines regularly. Approximately 55.3% of the cases checked their sugar level regularly, whereas 9.4% checked their sugar occasionally.

*(b) Blood Glucose Monitoring*. Of the cases, 25.2% did not have their glucose levels checked regularly. Of these, 31.8% (*n* = 49) checked their sugar levels at a health center; 22.7% (*n* = 35) measured their blood glucose at home, while 45.5% (*n* = 70) checked their blood glucose levels at health centers and at their homes.

*(c) Visit to Diabetes Clinics*. Of the cases, 92.7% (*n* = 140) attended diabetes clinics for follow-up, whereas 7.3% (*n* = 11) did not go for follow-up visits.

*(d) Diabetic Foot Care*. Approximately 58.2% (*n* = 92) of the cases were trained to take care of their feet, whereas 41.8% (*n* = 66) were not knowledgeable about diabetic foot care; 25.8% (*n* = 40) wore shoes designed for diabetic patients, while 74.2% (*n* = 115) did not.

*(e) Sleep Patterns of Diabetic Patients*. Of the cases, 74.1% (*n* = 117) reported having adequate sleeping hours, whereas 25.9% (*n* = 41) reported not having enough sleep. Approximately 15.1% (*n* = 19) of the cases had 2–4 hours of nightly sleep; 27.0% (*n* = 34) had 5-6 hours of nightly sleep; 57.9% (*n* = 73) had a nightly sleep duration of >7 hours at night.

*(f) Stress and Diabetes*. About 32.6% (*n* = 46) of the cases reported having psychological stress, whereas 67.4% (*n* = 95) did not report psychological stress.

By multivariate analysis, we found that cases were more likely to be older than 40 years (*P* < 0.0001), less educated (*P* = 0.05), married or divorced (*P* = 0.04), jobless/housewives (*P* < 0.0001), or current smokers (*P* = 0.002). They were also more likely to have salaries <7000 Saudi riyals (*P* = 0.01; Table 4).

## 4. Discussion

We found that male gender, age > 40 years, low educational attainment (illiterate or having completed primary school), salaries <7000 Saudi riyals, marital status (married or divorced), and smoking status (current smoker) were risk factors associated with DM2 in adult Saudi patients. It is probable that these individuals have the least information about dietary factors and the importance of self-care.

Regarding the nonmodifiable risk factors of DM (age, gender, and genetic factors), our findings that diabetic patients were more likely to be >40 years old and likely to have a family history of diabetes are similar to those reported earlier in the literature. In previous studies [16–18], it was reported that the prevalence of diabetes was higher in patients aged 45–64 years and in those who had a family history of DM [16–18]. Contrary to our finding, the authors [16, 18] reported that diabetes was predominant in women. However, our results are consistent with those of other authors who also reported diabetes to be more frequent in men [19–23]. In addition, we reported data from a different ethnic background (Saudi population). It is plausible that the differences in the prevalence of diabetes between men and women are due to the fact that nondiabetic men are generally more insulin-resistant than women [24].

In the current study, diabetic patients were more likely to be less educated; they were also more likely to have lower annual incomes. In a previous study [25], it was reported that low education and a higher annual income were associated with diabetes. Other authors [24] showed that the prevalence of diabetes was higher in women who had low incomes and a low socioeconomic status.

We demonstrated that the prevalence of diabetes was higher in married or divorced persons. Previous findings showed that marital status was not correlated with DM; however, differences in the prevalence of diabetes were slightly more noticeable in widowed or divorced persons [26]. Another study [27] showed that singlehood was associated with an increased risk of developing diabetes for women and an increased likelihood of death for men. Since it was not our aim to determine the association between marital status and diabetes, further studies are warranted to explore this factor.

Current smoking status is an independent modifiable risk factor for DM2 since it is associated with glucose intolerance, impaired fasting glucose, and, consequently, DM2. Our findings are consistent with those of other authors [28], who showed an association between diabetes and current smoking status. Therefore, an important measure of reducing the incidence of DM2 in the Saudi population may be to organize massive campaigns aimed at decreasing smoking across all age groups.

We showed that high BMI was significantly associated with diabetes, which might be because obesity enhances insulin resistance. Similar to our findings, previous studies [29, 30], including a study conducted on Saudi patients [30], also showed a direct relationship between BMI and diabetes. The increasing incidence of DM in the Saudi population has been linked to obesity, which is a consequence of major sociocultural and lifestyle changes. The promotion of fast foods, change in the traditional Saudi diet, both in quantity and quality, and physical inactivity are as a result of urbanization [31]. Hence, similar to other authors [28], we propose weight reduction and weight gain prevention as measures to control the rising incidence of DM. This is important because adult-onset diabetes, besides being linked to high BMI in men, is also associated with the duration of weight gain.

Our finding of an increased prevalence of hypertension in diabetic persons is similar to those reported in other studies [10, 31, 32]. It has been shown that although both hypertension and diabetes occur independently, they are known to exacerbate each other [32]. Furthermore, we found that a greater proportion of patients developed hypertension after diabetes diagnosis (43.1%) as compared with 23.1% of patients in whom hypertension was diagnosed prior to the diagnosis of diabetes. Since up to 75% of cardiovascular diseases in diabetic patients are attributed to hypertension, in persons with coexistent diabetes and hypertension, a more aggressive treatment and lifestyle management are recommended to reduce blood pressure to 140/90 mmHg [31]. In our sample, the mean blood pressure of diabetic hypertensive patients was 129.9/81.3 mmHg, which is within the range recommended by the American Diabetes Association [3]; however, most of the patients attended clinics for follow-up. The mean total cholesterol level of the cases in our study was 182.9 mg/dL, which is also within the recommended reference range for diabetic patients (<200 mg/dL) [3]. We attribute this finding to the use of lipid lowering agents among the cases in our study. Conversely, the mean LDL level of our patients was 111.4 mg/dL, which is above the range recommended for diabetic patients (<100 mg/dL) [3]. Hence, our cases have an increased risk to develop cardiovascular diseases. The mean HDL level among our cases was 47.6 mg/dL, which falls within the recommended range for men (>40 mg/dL) but not for women (recommended level, >50 mg/dL) [33]. Since diabetes is associated with multiple risk factors, diabetic hypertensive patients need more vigorous control of both lipids and glycosylated hemoglobin levels [32].

The second part of our study was to evaluate the status of diabetic patients regarding their diet, blood glucose monitoring habits, and awareness about diabetes care. Although we found that diabetic patients who visited clinics took their medicines regularly (93.6%), only half of the cases checked their glucose levels regularly and approximately 58.2% were knowledgeable about diabetic foot care. This is of concern because it is imperative for diabetic patients to be aware of the complications that may arise from diabetes, such as foot ulcers and possible amputation as a result of diabetic foot. We found that among patients who were aware about foot care, only 25.8% wore adequate shoes for diabetic patients. Hence, pharmacological control is not the only means of controlling diabetes, but education and self-awareness are also vital to prevent complications of diabetes.

Diabetic patients also need continuous counseling on the impact of consuming sugary foods and sweets as well as fried and fatty foods. In this study, we found that only 27.5% of diabetic patients did not consume sweets, and only 24.3% did not consume fried or fatty foods. Because the risk for diabetic patients to develop hypertension and associated cardiovascular diseases is doubled, additional measures are essential in these patients.

Massive educational and training programs aimed at counseling diabetic patients about all aspects of self care have to be initiated. Our data provide strong evidence to establish diabetic counseling for patients by nurses and physicians. Physicians have to be aware about these aspects and should be trained to counsel and guide diabetic patients. They should also be able to identify and counsel people who are at risk of developing diabetes. Besides, massive campaigns should be organized and aimed at educating the general population about the risk factors of DM. Young adults should also be informed that modernization, limited physical activity, and, consequently, obesity are triggering factors for the onset of diabetes.

## Figures and Tables

**Table 1:** Comparison of sociodemographic factors between cases and controls.

Variables	Controls (*n* = 128)		Cases (*n* = 159)		*P* value
	Count	%	Count	%	
Gender					
Male	19	15.1	60	38.0	<0.0001
Female	107	84.9	98	62.0	
Age (in years)					
<20	9	7.4	0	0.0	<0.0001
20–30	44	36.1	7	4.6	
31–40	22	18.0	10	6.6	
41–50	23	18.9	36	23.7	
51–60	18	14.8	50	32.9	
>60	6	4.9	49	32.2	
Educational level					
Illiterate	10	7.9	52	34.0	<0.0001
Primary	13	10.2	31	20.3	
Secondary	30	23.6	16	10.5	
Intermediate	18	14.2	24	15.7	
Bachelor degree	50	39.4	27	17.6	
Master degree	6	4.7	3	2.0	
Region					
Western Region	46	37.7	43	31.4	0.003
Eastern Region	7	5.7	31	22.6	
Southern Region	42	34.4	42	30.7	
Northern Region	11	9.0	14	10.2	
Other	16	13.1	7	5.1	
Family history of diabetes in blood relations					
Mother/father/brother/sister	75	58.6	114	71.7	0.020
Grandmother/father	40	31.3	27	17.0	0.005
Uncle/aunt	24	18.8	18	11.3	0.080
Occupation					
Employee	23	18.1	35	22.6	<0.0001
Jobless/housewife	59	33.9	84	54.2	
Retired	7	5.5	30	19.4	
Volunteer/student	54	425	6	3.26	
Salary (in Saudi riyals)					
1,000–2,000	15	12.9	21	14.2	<0.0001
2,000–5,000	28	24.1	69	46.6	
5,000–7,000	25	21.6	30	20.3	
7,000–10,000	17	14.7	17	11.5	
>10,000	31	26.7	11	7.4	
Marital status					
Single	36	29.8	8	5.2	<0.0001
Married	71	58.7	113	73.4	
Divorced/widowed	14	11.6	33	21.4	
Nature of Job					
Physically active work	12	9.4	23	14.5	0.300
Physically inactive work	11	8.6	12	7.5	
Not applicable	105	82	124	78	
Do you do household chores?					
Yes	74	58.7	68	46.9	0.060
Hypertension					
Yes	27	21.6	77	49.7	<0.0001
No	98	78.4	78	50.3	

**Table 2:** Univariate analysis showing crude association of sociodemographic factors with diabetes mellitus type 2.

Variables	Unadjusted OR (95% CI)	*P* value
Gender		
Male	3.45 (1.92–6.18)	<0.0001
Female	Reference	
Age		
<40	Reference	<0.0001
41–50	6.91 (3.29–14.51)	
51–60	12.25 (5.77–26.03)	
>60	36.03 (13.28–97.73)	
Educational level		
Illiterate	10.4 (2.22–48.62)	<0.0001
Primary	4.77 (1.03–22.02)	
Secondary	1.07 (0.23–4.84)	
Intermediate	2.67 (0.59–12.13)	
Bachelor degree	1.08 (0.25–4.66)	
Master degree	Reference	
Region		
Western Region	Reference	0.003
Eastern Region	4.74 (1.89–11.88)	
Southern Region	1.07 (0.59–1.94)	
Northern Region	1.36 (0.56–3.32)	
Other	0.47 (0.18–1.25)	
Occupation		
Employee	Reference	<0.0001
Volunteer/student	0.07 (0.03–0.2)	
Jobless/housewife	1.28 (0.68–2.44)	
Retired	2.82 (1.06–7.48)	
Salary (in Saudi riyals)		
1,000–2,000	3.95 (1.52–10.25)	<0.0001
2,000–5,000	6.94 (3.07–15.71)	
5,000–7,000	3.38 (1.42–8.06)	
7,000–10,000	2.82 (1.08–7.37)	
>10,000	Reference	
Marital status		
Single	Reference	<0.0001
Married	7.16 (3.15–16.29)	
Divorced/widowed/separated	10.61 (3.95–28.51)	
Family history of diabetes in blood relatives		
Mother/father/brother/sister	1.79 (1.09–2.93)	0.020
Grandmother/father	0.45 (0.26–0.79)	0.005
Uncle/aunt	0.55 (0.29–1.07)	0.080
Nature of Job		
Physically active work	1.76 (0.6–5.15)	0.300
No physically active work	Reference	
Do you do household chores?		
Yes	Reference	0.060
No	1.93 (1.11–3.35)	
Sometimes	1.14 (0.58–2.26)	
Do you have a servant at home?		
Yes	0.87 (0.53–1.43)	0.580
No	Reference	
Compared to others of your age, you can say you are		
More active	0.74 (0.42–1.32)	0.590
Less Active	0.91 (0.51–1.63)	
No difference	Reference	
Currently smoking		
Yes	3.97 (1.58–9.98)	0.003
No	Reference	
Hypertension		
Yes	3.58 (2.11–6.09)	<0.0001
No	Reference	
BMI (in kg/m^2^)		
<25	Reference	<0.001
25–29.9	2.32 (1.19–4.49)	
≥30	7.64 (3.33–17.54)	

BMI: body mass index; CI: confidence interval; OR: odds ratio.

**Table 3:** Clinical finding in control and cases.

Parameter	Mean	SD	*n *	Mean	SD	*n *
Weight	65.2	16.6	92	75.4	16.5	88
Height	158.7	9.9	85	154.6	30.8	69
Blood glucose level (last reading) Fasting blood sugar (mg/dL)	—	—	—	137.4	71.1	80
Blood glucose level (last reading) Postprandial blood sugar (mg/dL)	—	—	—	143.5	95.3	35
Systolic BP (mmHg)	119.9	18.3	61	129.9	20.3	76
Diastolic BP (mmHg)	77.2	10.1	61	81.3	23.5	77
Cholesterol (mg/dL)	127.4	69.8	9	182.9	37.6	30
Total cholesterol (mg/dL)	142.3	63.3	10	177.1	83.6	24
HDL (mg/dL)	40.7	7.3	12	47.6	27.2	42
LDL (mg/dL)	135.7	53.3	14	111.4	36.0	41

BP: blood pressure; HDL: high-density lipoprotein; LDL: low-density lipoprotein; SD: standard deviation.

**Table 4:** Multivariate logistic regression model for association of risk factors with diabetes among adults.

Variables	Cases (%)	Controls (%)	Adjusted OR	*P* value
Age (in years)				
<40	61.5	11.2	Reference	<0.0001
41–50	23.7	18.9	14.84 (3.92–56.23)	
51–60	32.9	14.8	24.59 (5.88–102.85)	
>60	32.2	4.9	44.91 (8.12–248.49)	
Educational level				
Illiterate	34.0	7.9	6.04 (0.47–78.49)	0.050
Primary	20.3	10.2	8.59 (0.7–104.68)	
Secondary	15.7	14.2	1.11 (0.1–12.01)	
Intermediate	10.5	23.6	3.65 (0.33–40.51)	
Bachelor degree	17.6	39.4	4.74 (0.53–42.41)	
Master degree	2.0	4.7	Reference	
Occupation				
Employee	22.6	18.1	Reference	<0.0001
Volunteer/student	3.8	42.5	0.01 (0.00–0.06)	
Jobless/housewife	54.2	33.9	0.37 (0.1–1.34)	
Retired	19.4	5.5	0.22 (0.05–1.05)	
Salary (in Saudi riyals)				
1,000–2,000	14.2	12.9	9.97 (1.53–64.79)	0.010
2,000–5,000	46.6	24.1	9.24 (2.17–39.37)	
5,000–7,000	20.3	21.6	2.4 (0.56–10.31)	
7,000–10,000	11.5	14.7	7.32 (1.39–38.46)	
>10,000	7.4	26.7	Reference	
Marital status				
Single	5.2	29.8	Reference	0.040
Married	73.4	58.7	0.15 (0.03–0.79)	
Divorced/widowed	21.4	11.6	0.09 (0.01–0.61)	
Currently smoking				
Yes	16.4	4.7	13.74 (2.59–72.85)	0.002
No	83.6	95.3	Reference	
